# Long-term impact of sleeve gastrectomy on serum uric acid levels

**DOI:** 10.55730/1300-0144.5574

**Published:** 2022-09-21

**Authors:** Birkan BİRBEN, Gökhan AKKURT, Mesut TEZ, Barış Doğu YILDIZ

**Affiliations:** Department of Surgery, Ankara City Hospital, Ankara, Turkey

**Keywords:** Uric acid level, sleeve gastrectomy, obesity, body mass index, excess weight loss

## Abstract

**Background/aim:**

Obesity is a risk factor for hyperuricemia. Bariatric surgery is an effective treatment method for metabolic control. The aim of our study was to evaluate the correlation between the preoperative and postoperative serum uric acid levels with body mass index, body weight, and excess weight loss in patients that underwent sleeve gastrectomy.

**Materials and methods:**

A total of 164 patients that underwent laparoscopic and open sleeve gastrectomy were evaluated in terms of the demographic characteristics, preoperative body weight, body mass index and serum uric acid levels, and postoperative body weight, body mass index, excess weight loss and serum uric acid levels at the first, third, sixth, 12^th^, 18^th^ and 24^th^–36^th^ months.

**Results:**

There was a statistically significant increase in serum uric acid levels in the first postoperative month (p = 0.000). The patients with a high preoperative body mass index were found to have a lower excess weight loss in the first postoperative month, which was statistically significant (p = 0.000, R = −0.474). Serum uric acid levels were also positively correlated with body weight at the third and sixth postoperative months.

**Conclusion:**

The weight loss associated with sleeve gastrectomy is the main reason for the reduced postoperative serum uric acid levels.

## 1. Introduction

Hyperuricemia is characterized by elevated serum uric acid (SUA) levels secondary to changes in purine metabolism [1]. Uric acid is mainly produced in liver and small intestine, and excreted from kidney and gastrointestinal tract [2]. Obesity is a risk factor for hyperuricemia and was associated with insulin resistance [3,4]. Increased concentration of uric acid in serum leads to the formation of uric monosodium crystals, for which the threshold is 7 mg/dL [2]. Bariatric surgery is an effective treatment option for metabolic control in morbidly obese cases, and one of the most common bariatric methods is sleeve gastrectomy. The aim of this study was to evaluate the correlation of preoperative and postoperative SUA levels with body mass index (BMI), body weight (BW), and excess weight loss (EWL) in patients who underwent sleeve gastrectomy.

## 2. Materials and methods

### 2.1. Patient data

After obtaining approval from the local ethics committee (approval number: E-19-2576), 303 patients that underwent laparoscopic and open-sleeve gastrectomy by a single surgeon between January 2010 and October 2018 were retrospectively evaluated. The patients with incomplete data, those with elevated creatinine levels, and those receiving diuretic, aspirin, or corticosteroid treatment were excluded. As a result, the records of 164 patients were examined in terms of demographic characteristics, preoperative body weight, BMI and SUA levels and postoperative body weight, BMI, EWL, and SUA levels at the first, third, sixth, 12^th^, 18^th^, and 24^th^-36^th^ months. The correlation of the SUA levels with BMI, EWL, and body weight was investigated. For hyperuricemia, 7 mg/dL was accepted as the threshold according to the plasma range recommended by the laboratory, and the patients were also examined in two different groups depending on their preoperative SUA levels being above or below 7 mg/dL.

### 2.2. Statistical analysis

The SPSS 15.0 (SPSS Inc., Chicago, USA) statistics software was used for statistical analysis. The Shapiro-Wilk test was used to assess the normality of data distribution. Continuous data were presented as the mean value ± standard deviation. Differences in continuous variables were analyzed using the Mann-Whitney U test. Categorical variables were analyzed using chi-square tests. Correlation analysis of the continuous variables was performed by Pearson’s correlation test. A p-value of less than 0.05 was considered significant.

## 3. Results

Of the 164 patients included in the study, 138 were female and 26 were male. The mean preoperative BMI and body weight were 49.61 ± 7.37 kg/m^2^ and 155.17 ± 25.11 kg, respectively for men and 49.14 ± 6.66 kg/m^2^ and 129.68 ± 18.18 kg, respectively for women. The mean preoperative uric acid level of all patients was 5.825 ± 1.2 mg/dL. There was a statistically positive correlation between the preoperative BMI and preoperative serum uric acid values (p = 0.017, R = 0.187). Similarly, the preoperative serum uric acid values were statistically positively correlated with the preoperative body weight (p = 0.002, R = 0.243). In the first postoperative month, 70.7% of the patients with an increase in serum uric acid values had increase in creatinine levels and it was observed that changes in SUA values correlated with creatinine levels (p = 0.000, R = 0.377). However, creatinine values were within the normal range. Our institutional policy requires measurement of BMI rather than waist circumference. The mean BMI of our patients was 49.44 ± 6.76 kg/m2. There is a strong correlation between BMI and waist circumference. So all of our patients who were classified as obese according to BMI fulfill one item for diagnosis of metabolic syndrome. Metabolic syndrome was present in 43 (26.7%) of the patients. There was no metabolic syndrome in 118 (73.3%) patients. Data of 3 patients could not be reached. Mean uric acid values of patients with and without metabolic syndrome were 6.01 ± 1.14, 5.77 ± 1.35, respectively. The uric acid increase was not statistically significant when the metabolic syndrome group and nonmetabolic syndrome group were compared (p = 0.304). The comparison of the patients with preoperative hyperuricemia and normal uric acid levels is given in Table 1.

Of the patients with an SUA value of less than 7 mg/dL, 127 were female and 12 were male. The mean age was 40 ± 11 years. The mean preoperative BMI, body weight, and SUA levels were 48.9 ± 6.45 kg/m^2^, 131.64 ± 20.05 kg and 5.44 ± 0.98 mg/dL, respectively. In the group with an SUA of greater than 7 mg/dL, there were 11 female and 14 male patients with a mean age of 38 ± 9 years. In this group, the mean preoperative BMI, body weight and SUA values were 51.91 ± 7.99 kg/m^2^, 147.63 ± 24.53 kg, and 7.91 ± 0.74 mg/dL, respectively. There was not a statistically significant difference between the groups with an SUA level of above and below 7 mg/dL in terms of preoperative BMI (p = 0.098). However, the patients with a high SUA value had a statistically significant higher body weight (p = 0.001). Similarly, while no significant difference was observed according to age (p = 0.549), the SUA value was significantly higher in patients with male patients (p = 0.000).

In the first postoperative month, the mean SUA value of all patients was 6.6027 ± 1.88 mg/dL, which was statistically significantly higher compared to the mean preoperative value (p = 0.000). Changes in SUA values did not significantly correlated with EWL, BW, and BMI in the first postoperative month. In patients with a mean preoperative SUA of below 7 mg/dL, the mean SUA at the first postoperative month was calculated as 6.30 ± 1.8 mg/dL, which indicated a statistically significant increase (p = 0.000). For the group with a preoperative SUA level of above 7 mg/dL, the mean SUA was determined as 8.26 ± 1.69 mg/dL in the postoperative first month, and the increase was not statistically significant (p = 0.683). The patients with a higher preoperative BMI had statistically significantly lower EWL values at the first postoperative month (p = 0.000, R = −0.474). Table 2 shows the statistical correlation of the postoperative SUA values with EWL, BMI, and body weight.

The SUA values were positively correlated with body weight in the third and sixth postoperative months. The preoperative and postoperative changes in SUA and body weight are given in Figure.

## 4. Discussion

Obesity is an important health problem affecting the quality of life and leading to the development of comorbidities. Hyperuricemia is one of the problems caused by these comorbidities. In the literature, it was reported that in obese patients, sleeve gastrectomy reduced EWL by 60%–65% and controlled comorbidities at a rate of 45%–95% over a three-year follow-up [5]. Our study differs from the previous works in that it presented the correlation curve of SUA, EWL, BMI, and body weight for the preoperative period and each follow-up assessment in the postoperative period over three years.

Menenakos et al. reported that the preoperative SUA concentration was positively correlated with the preoperative BMI [2]. In a similar study, Rathmann et al. showed that the SUA concentration increased as BMI increased [6]. Similarly, in the current study, the preoperative SUA concentration was correlated with the preoperative BMI and preoperative body weight. In addition, it was observed that preoperative hyperuricemia was significantly more common in patients with higher preoperative body weight and male sex. The higher incidence of hyperuricemia in males was associated with their higher body weight. Menenakos et al. emphasized that according to the recent literature, preoperative BMI did not predict postoperative weight loss, and in their own study, the authors also revealed that preoperative BMI was not correlated with the percentage of total EWL in the first month and year [2]. In the current study, the preoperative BMI had a negative correlation with EWL in the first postoperative month. Nielsen et al. reported an increase in SUA levels in the early period after bariatric surgery [7]. In our study, a statistically significant increase was observed in the SUA levels in the first postoperative month. The increase in SUA in the postoperative first month was statistically significant in patients with normal preoperative SUA levels, but not those with preoperative hyperuricemia. Schiavo et al. found that the consumption of concentrated food products with high purine content, such as meat broth increased the level of SUA [8]. Elevated SUA concentrations negatively affect the transport of glucose by suppressing nitric oxide production in the body and causes renal vasoconstriction and tubulointersitial damage. In addition, uric acid accumulated in the joints is crystallized and leads to the development of gout [9]. These effects were not observed in any of our patients with elevated SUA. In our study it was observed that changes in SUA values correlated with creatinine levels in the first postoperative month and none of our patients experienced gout attacks before or after surgery. This might mean that increased serum uric acid levels are transient and do not cause permanent alterations in tissue uric acid binding. Therefore, drug therapy for uric acid metabolism was not applied. Maglio et al. reported that bariatric surgery prevented hyperuricemia in obese patients and decreased SUA levels in the long term [10]. In a similar study, Romero-Talamas et al. determined that SUA levels were significantly reduced at the 13^th^ postoperative month after bariatric surgery [11]. In our long-term follow-up, we observed that the SUA level which increased in the first postoperative month showed a decline in the third and sixth months in correlation with body weight but did not show such a correlation from the 12^th^ to the 36^th^ months.

In conclusion, although the preoperative SUA level is correlated with the preoperative BMI and body weight, the primary factor in hyperuricemia is the total body weight. The main reason for the reduced postoperative SUA value associated with sleeve gastrectomy is that this surgery results in a decrease in body weight in long term. Based on our results, we conclude that hyperuricemia that develops early in the postoperative period after sleeve gastrectomy is spontaneously resolved and does not require medication.

## Figures and Tables

**Figure:**
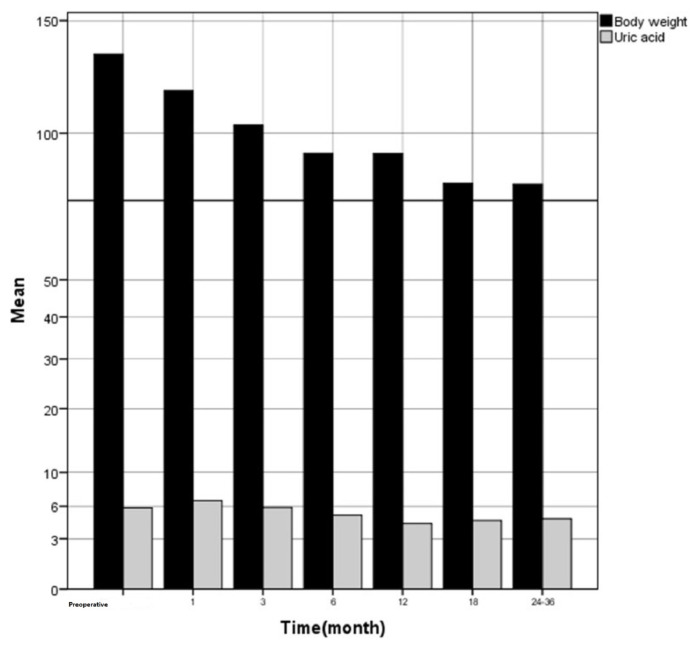
Changes in serum uric acid and body weight.

**Table 1:** Comparison of the patients according to the preoperative SUA levels.

n = 164	SUA < 7mg/dL	SUA ≥ 7mg/dL
Sex (F/M)	127/12	11/14
Age (mean)/years	40 ± 11	38 ± 9
BMI (kg/m^2^)	48.9 ± 6.45	51.91 ± 7.99
Body weight (kg)	131.64 ± 20.05	147.63 ± 24.53
SUA (mean) (mg/dL)	5.44 ± 0.98	7.91 ± 0.74

SUA; serum uric acid, F; female, M; male, BMI; body mass index

**Table 2:** Changes in the postoperative SUA values according to EWL, BMI, and body weight.

	EWL/SUA p-value	Weight/SUA p-value	BMI/SUA p-value
First month	0.088	0.208	0.457
Third month	0.315	0.009 R = 0.326	0.112
Sixth month	0.801	0.006 R = 0.340	0.656
12th month	0.570	0.929	0.663
18th month	0.645	0.669	0.966
Second to third year	0.459	0.218	0.271

SUA; serum uric acid, EWL; excess weight loss, BMI; body mass index

## References

[1] Zhang Y Wang J Sun X Cao Z Xu X Laparoscopic sleeve gastrectomy versus laparoscopic Roux-en-Y gastric bypass for morbid obesity and related comorbidities: a meta-analysis of 21 studies Obesity Surgery 2015 25 1 19 26 10.1007/s11695-014-1385-9 25092167

[2] Menenakos E Doulami G Tzanetakou IP Natoudi M Kokoroskos N The use of serum uric acid concentration as an indicator of laparoscopic sleeve gastrectomy success International Surgery 2015 100 1 173 179 10.9738/INTSURG-D-13-00186.1 25594659 PMC4301285

[3] Yadav D Lee ES Kim HM Lee EY Choi E Hyperuricemia as a potential determinant of metabolic syndrome Journal of Lifestyle Medicine 2013 3 2 98 106 26064845 PMC4390742

[4] Katsogridaki G Tzovaras G Sioka E Perivoliotis K Zachari E Hyperuricemia and acute gout after laparoscopic sleeve gastrectomy Clinical Obesity 2019 9 2 e12296 10.1111/cob.12296 30815983

[5] Shi X Karmali S Sharma AM Birch DW A review of laparoscopic sleeve gastrectomy for morbid obesity Obesity Surgery 2010 20 8 1171 1177 10.1007/s11695-010-0145-8 20379795

[6] Rathmann W Haastert B Icks A Giani G Roseman JM Ten-year change in serum uric acid and its relation to changes in other metabolic risk factors in young black and white adults: the CARDIA study European Journal of Epidemiology 2007 22 7 439 445 10.1007/s10654-007-9132-3 17484024

[7] Nielsen SM Bartels EM Henriksen M Wæhrens EE Gudbergsen H Weight loss for overweight and obese individuals with gout: a systematic review of longitudinal studies Annals of the Rheumatic Diseases 2017 76 11 1870 1882 10.1136/annrheumdis-2017-211472 28866649 PMC5705854

[8] Schiavo L Favrè G Pilone V Rossetti G De Sena G Low-purine diet is more effective than normal-purine diet in reducing the risk of gouty attacks after sleeve gastrectomy in patients suffering of gout before surgery: a retrospective study Obesity Surgery 2018 28 5 1263 1270 10.1007/s11695-017-2984-z 29058239

[9] Nakagawa T Kang D Feig D Sanchez-Lozada L Srinivas T Unearthing uric acid: an ancient factor with recently found significance in renal and cardiovascular disease Kidney International 2006 69 10 1722 1725 10.1038/sj.ki.5000391 16598194

[10] Maglio C Peltonen M Neovius M Jacobson P Jacobsson L Effects of bariatric surgery on gout incidence in the Swedish Obese Subjects study: a non-randomised, prospective, controlled intervention trial Annals of the Rheumatic Diseases 2017 76 4 688 693 10.1136/annrheumdis-2016-209958 28076240 PMC5530073

[11] Romero-Talamás H Daigle CR Aminian A Corcelles R Brethauer SA The effect of bariatric surgery on gout: a comparative study Surgery for Obesity and Related Diseases 2014 10 6 1161 1165 10.1016/j.soard.2014.02.025 24935177

